# Coil Inductance Model Based Solenoid on–off Valve Spool Displacement Sensing via Laser Calibration

**DOI:** 10.3390/s18124492

**Published:** 2018-12-18

**Authors:** Hao Tian, Yuren Zhao

**Affiliations:** Department of Mechanical Engineering, Dalian Maritime University, Dalian 116026, China; yuren1819@dlmu.edu.cn

**Keywords:** on–off solenoid, air gap, spool displacement sensing, solenoid inductance, laser triangulation sensor

## Abstract

Direct acting solenoid on–off valves are key fluid power components whose efficiency is dependent upon the state of the spool’s axial motion. By sensing the trajectory of the valve spool, more efficient control schemes can be implemented. Therefore, the goal of this study is to derive an analytical model for spool displacement sensing based on coil inductance. First, a mathematical model of the coil inductance as a function of air gap width and lumped magnetic reluctance is derived. Second, to solve the inductance from coil current, an optimization to obtain an initial value based on physical constraints is proposed. Furthermore, an experiment using a laser triangulation sensor is designed to correlate the magnetic reluctance to the air gap. Lastly, using the obtained empirical reluctance model to eliminate unknowns from the proposed air gap-inductance model, the model in atmosphere or hydraulic oil environments was tested. Initial results showed that the proposed model is capable of calculating the spool displacement based on the coil current, and the estimation errors compared to the laser measurement are within ±7% in air environment.

## 1. Introduction

Solenoid on–off valves are key control elements in a fluid power system. Especially, the direct acting single-stage types are widely implemented in the heavy industry sectors due to their robustness, contamination resistance, relative fast response time, and remote controllability. A direct acting solenoid on–off valve consists of an electromagnetic driving coil, a ferrous plunger that translates along the axis of the coil, and a sliding valve spool driven by the plunger with machined orifice. An air gap usually presents along the coil axis between the plunger and armature when the valve is deenergized. When energized, the electromagnetic force pulls in the plunger eliminating the gap, causing the valve to transit from one flow state to another. According to Bernoulli’s Equation, the flow through a variable orifice is governed by the Reynolds number dependent discharge coefficient and the pressure differential across the valve. Since the valve state is directly dependent upon the spool axial displacement, it is therefore of great incentive to sense the position of the valve spool, for better energy loss evaluation and further improvement on the valve-controlled system efficiency.

To measure the displacement of the valve spool, traditionally there are two approaches: the contact and noncontact ones. In contact approaches, displacement sensors are mechanically connected to the valve spool for position measurement. For example, the D638 series direct acting servo valve from Moog [1] and D1FC series direct operated proportional valve from Parker [2] both employ the idea of spool position feedback by coupling a position sensor (e.g., usually a linear variable differential transformer—LVDT) to the valve spool for closed loop control. By doing so, the volumetric flow rate through the valve can be simply but precisely regulated by a reference input. However, since the solenoid valve is a spring-mass-damper system, attaching a sensor to the valve spool essentially alters the natural frequency, reducing the responsiveness of the valve. As argued by Tu et al. [3], the switching frequency of the valve determines the overall efficiency of a digital fluid power system, a slower valve means more flow energy is wasted as generated heat.

Mechanically coupling a displacement sensor to the spool is straightforward, but in the research of fast response on–off valves, it is usually structurally or dynamically infeasible, thus noncontact measurement methods have been developed. Mahrenholz et al. [4] developed a disc-type high-speed solenoid on–off valve, and due to the complexity of the mechanical structure, the flow pressure at the valve outlet was used as the reference to verify the on–off action of the valve. Holland [5] also used the pressure signal to indicate the on–off valve state when used in a digital pump-motor. While the pressure signal is readily available in most fluid power systems, using the threshold pressure to indicate spool extreme positions is easy to implement. Due to the inherent nonlinearity between the pressure drop and valve open area as stated in the orifice discharge flow equation, the displacement sensitivity drops drastically as the valve starts to open, resulting in difficulties in measuring the complete time history of the spool position other than key timing points. Compared to the pressure indicator, the electromagnetic characteristics of the solenoid coil, such as magnetic flux, eddy current, coil current, or coil inductance, could have better displacement sensitivity over the entire range of spool travel and are developed to locate the spool position. Romer et al. [6] installed permanent magnet on the valve poppet and measured its position using a Hall effect transducer. Winkler et al. [7] used the eddy current sensed from the shim ring, a structure attached to the valve poppet, to measure the poppet position without attaching additional contact-type sensors. However, both require modification to the spool. For non-invasive, non-contact sensing of the valve spool position, the coil current or its derived inductance could be a prospective candidate for the spool displacement sensing, and laser triangulation sensors are generally utilized as a reference signal to calibrate the spool position [8]. Vaughan et al. [9] developed an electromagnetic model for a proportional solenoid valve, and the model indicates the nonlinear time varying characteristics of the coil inductance as the spool translates. Yudell et al. [10] modeled the electric and magnetic circuit of a solenoid on–off valve, and used two particular points with certain rate changes in the coil current to represent the two extreme positions of the valve spool. However, the initial value of inductance is difficult to obtain due to strong nonlinearity of the electromagnetic system, and usually an observer needs to be constructed [11]. Eyabi et al. [12] designed a nonlinear spool displacement observer for closed-loop stroke control of a solenoid actuator based on the coil current. Gluck et al. [13] designed a displacement observer based on the inductance of a magnetic levitation system and positionally controlled the magnetically levitated object. Braun et al. [14] proposed and tested a spool displacement observer based on coil reluctance and inductance for a proportional solenoid, and more recently, using only the coil current and voltage to estimate the poppet position of an on–off solenoid [15]. Wu et al. [16] implemented a sliding-mode observer to achieve sensor-less spool position estimation for a high-speed solenoid on–off valve. To sense the spool displacement, there are also reported works by superposition of a sinusoidal signal over the coil driving current [17], or by separating the AC (alternative current) and DC (direct current) components of the driving signal [18], both targeted to estimate the solenoid inductance with induced or localized electrical circuit dynamics, then fitted to spool displacement.

In most of the methods above, a solution to the dynamics of an observer or additional superimposed signal decoupling and processing is usually mandatory, which would require significant computational effort if the control is in “real time” or at high frequencies. Thus, to relax the hardware requirement for more portable and integrated hydraulic components, the motivation of this work is to develop an analytical model for non-contact spool position sensing of an industrial on–off solenoid valve via calculated inductance from coil current. Additionally, to accurately obtain the magnitude of the initial inductance, an optimization utilizing the inductance-resistor circuit property is also proposed.

To derive the proposed coil inductance-spool displacement (air gap) model, the paper is organized as follows. Section 2 focuses on the efforts of electromagnetic modeling of actuation coil current and the relationship between coil inductance and spool position. An experimental test bed is set-up and laser triangulation sensor-based valve spool calibration is performed in Section 3. Finally, the verification of position measurement results when the valve is operated under different conditions is performed and discussed in Section 4 and Section 5. Initial results of the use of coil inductance can achieve spool displacement measurement with overall estimation error less than ±7%.

## 2. On–off Solenoid Electromagnetic Model

**Figure 1:**
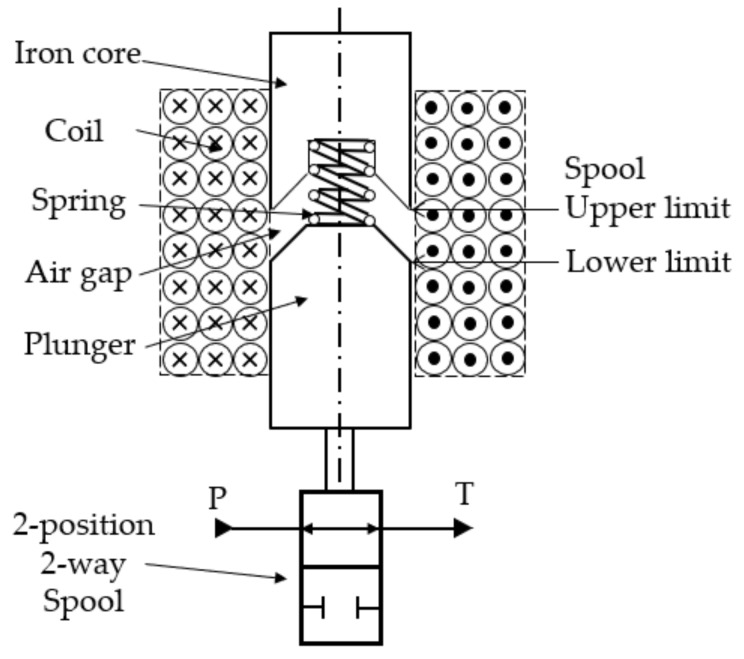
Structure diagram of a SV08-25 solenoid-operated on–off valve.

The research object is a two-way, two-position solenoid on–off valve (Model SV08-25, manufactured by Hydraforce Inc., Lincolnshire, IL, USA), commonly found in industrial fluid power systems (e.g., excavators, tractors, etc.). The rated working pressure is 20 MPa, and the rated maximum flow is 9.5 L/min [19]. Its structure is shown in Figure 1. The P port is the oil inlet and the T port is the outlet. When the driving coil is powered off, the armature assembly is subjected to the spring preload force, stopped on the bottom end of its stroke, thus the P port and T port are connected. When the coil is energized, generated magnetic field potential attracts the plunger and overcomes the spring force, moving the plunger from the bottom to the top end of the spool stroke, which disconnects the T port from the P port.

### 2.1. Electromagnetic Model

**Figure 2:**
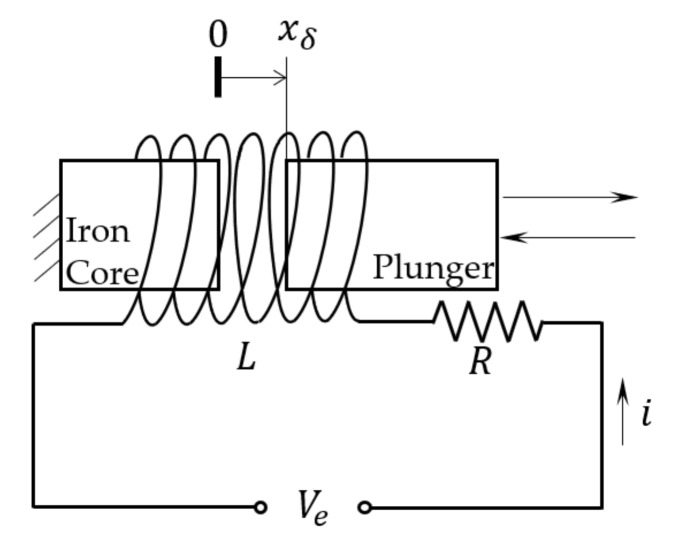
Diagram of valve’s driving coil.

The solenoid coil, plunger, and spool constitute an electric circuit with time varying inductance but constant resistance as shown in Figure 2. According to Kirchhoff’s law of voltage, the voltage applied to the coil is equal to the summation of voltage drop across the equivalent coil resistance and the electromotive potential caused by changes in magnetic flux:

(1) $$V_{e}=iR+N\frac{d\Phi }{dt}\;,$$

where $V_{e}$ is the excitation voltage, i is the current in the coil, R is the equivalent resistance of the coil, N is the number of coil turns, and Φ is the average magnetic flux of each turn of the coil.

According to the classical electromagnetic theory, the constitutive relationship between the magnetic flux linkage (ψ), the magnetic flux (Φ), and the inductance (L) is

(2) ψ=NΦ=Li,

Combine Equations (1) and (2) and the coil circuit equivalent model can be obtained:

(3) $$V_{e}=iR+i\frac{dL}{dt}+L\frac{di}{dt}\;,$$

Equation (3) is a first order ordinary differential equation (ODE) of L about i, indicating that the by measuring the coil current, the inductance of the coil can be solved. And since the plunger’s reciprocating motion creates a variable width air gap, the inductance of the solenoid fluctuates. As a result, the spool’s (same as the plunger’s) displacement can be obtained from the inductance information.

### 2.2. Spool Displacement Function 

**Figure 3:**
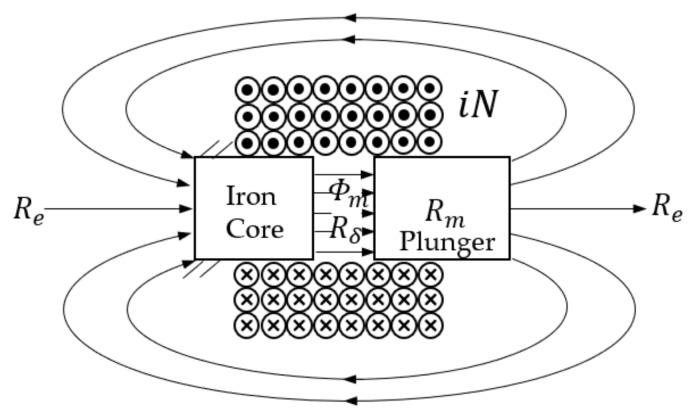
Diagram of magnetic circuit of the on–off solenoid.

The fixed iron core, the reciprocating plunger, and the coil formed an on–off solenoid. Since the on–off solenoid in this study does not feature a typical torus iron core as in other research [20], which ferrous material structured in a nearly closed loop would confine most of the magnetic flux within, the magnetic flux line in this work would extend from the pole on the plunger back to the one on the iron core, as shown Figure 3. According to Hopkinson’s law (similar to Ohm’s law for an electrical circuit), the magnetic circuit can be modeled as

(4) $$iN=\Phi_{m}\left(R_{\delta }+R_{m}+R_{e}\right),$$

where $\Phi_{m}$ is the magnetic flux in the magnetic circuit, $R_{\delta }$ is the magnetic reluctance of working air gap, $R_{m}$ in the core and plunger, and $R_{e}$ elsewhere.

Industrial on–off valves are usually made of soft magnetic materials with large saturation magnetic induction and small remanence, and its magnetic permeability is much larger than that of the air. In this case, $R_{m}\approx 0$, therefore Equation (4) can be simplified as:

(5) $$iN≅\Phi_{m}\left(R_{\delta }+R_{e}\right),$$

The magnetic reluctance of the air gap is

(6) $$R_{\delta }=\frac{x_{\delta }}{\mu_{0}S}\;,$$

where $x_{\delta }$ is the air gap height, $\mu_{0}$ is the magnetic permeability in vacuum, and S is the effective air gap area.

The derivation of the spool displacement function started from the combination of Equations (2) and (5). The resulting Equation (7) indicates that the static inductance of the solenoid valve is a function only of the coil turns and the total magnetic reluctance:

(7) $$L=\frac{N^{2}}{R_{\delta }+R_{e}},$$

Substitute $R_{\delta }$ into Equation (7), resulting in the definition of the on–off valve solenoid’s inductance, which is a function of air gap width (Equation (8)). Since air gap variation changes the plunger’s position within the solenoid, so does the characteristics of the magnetic circuit. As a result, the lumped reluctance ($R_{e}$) can be considered also affected by $x_{\delta }$:

(8) $$L\left(x_{\delta },t\right)=\frac{\mu_{0}SN^{2}}{x_{\delta }\left(t\right)+\mu_{0}SR_{e}\left(x_{\delta }\right)}\;,$$

Equation (8) defines the relationship between $L\left(x_{\delta },t\right)$ and $x_{\delta }\left(t\right)$, and by solving the inverse, $L^{-1}\left(x_{\delta },t\right)$, $x_{\delta }$ at time t can be determined. However, an obstacle preventing explicit inversion is that $R_{e}$ is also a function of $x_{\delta }$. To address this issue, an experiment is designed to resolve the mathematical relationship between $R_{e}$ and $x_{\delta }$ in Section 4.3.

### 2.3. Numerical Solution Methods

According to Equation (8), the time varying inductance in Equation (3) is the key to solving the spool displacement. The magnitude of L can be calculated by solving the (ODE) through substitution of the standard fixed-step forward difference approximation of the inductance’s and electric current’s time derivative (i.e., $\frac{dL}{dt}=\frac{L_{k+1}-L_{k}}{∆t}$, $\frac{di}{dt}=\frac{i_{k+1}-i_{k}}{∆t}$) into Equation (3):

(9) $$L_{k+1}=\frac{V_{e}{}_{k+1}-i_{k+1}R-L_{k}\frac{i_{k+1}-i_{k}}{∆t}}{i_{k+1}}∆t+L_{k},$$

where k denotes the iterative time step, and ∆t is the time step length, in this work, ∆t= 1 × 10^−4^ s. The convergence of the algorithm with respect to ∆t is discussed in Appendix A.

The coil parameters, coil current, and the circuit resistance can be easily measured, so the coil inductance can be calculated at each time step using Equation (9). However, the implicit relationship of Equation (8) between inductance and air gap width leaves two major obstacles in solving $L^{-1}\left(x_{\delta },t\right)$ directly. First, to solve Equation (3) or (9), initial condition about $L_{0}$ is necessary but unknown. Second, it is difficult to solve $x_{\delta }$ from L analytically without knowledge of $R_{e}$. To tackle the first challenge, an optimization is performed to find out the required $L_{0}$ by exploiting the physical meaning of Equation (8), since the derivation of inductance w.r.t. air gap is

(10) $$\frac{\partial L}{\partial x_{\delta }}=-\frac{\mu_{0}SN^{2}\left(1+\mu_{0}S\frac{dR_{e}}{dx_{\delta }}\right)}{{\left(x_{\delta }+\mu_{0}SR_{e}\right)}^{2}},$$

when the spool is in a fixed position at steady state, both $x_{\delta }$ and $R_{e}$ should be constants, that gives

(11) $$∆L=-\frac{\mu_{0}SN^{2}}{{\left(x_{\delta }+\mu_{0}SR_{e}\right)}^{2}}∆x_{\delta }=0,$$

Thus, the objective function is to find $L_{0}$ that satisfies the optimization

(12) $$\min{\left\|\frac{\partial \hat{L}\left(L_{0}^{*},t\right)}{\partial t}\right\|}_{t\in \left(0,t_{s}\right]},$$

where $\hat{L}\left(L_{0}^{*},t\right)$ denotes the calculated inductance as the spool is held stationary with a given initial condition $L_{0}^{*}$, and $t_{s}$ is the length of the sample time. 

The optimization in Equation (12) can be done by grid search. The lower bound is defined by the physical meaning of inductance, which demands $L_{0}^{L}>0$. For the upper bound, according to Equation (8), the inductance is inversely proportional to the sum of the magnetic reluctance of the air gap and the ambient, its magnitude should be less than $L_{0}^{U}<\frac{\mu_{0}SN^{2}}{x_{\delta }\left(t\right)}$. Then a grid search can be performed over $\left(L_{0}^{L},L_{0}^{U}\right)$ to find $L_{0}$.

For the second challenge, $L^{-1}\left(x_{\delta },t\right)$ is solved by experimental identification of $R_{e}\left(x_{\delta }\right)$, the experiment design is shown in the section that follows.

## 3. Experiment Design

### 3.1. Optical Measurement Principle

**Figure 4:**
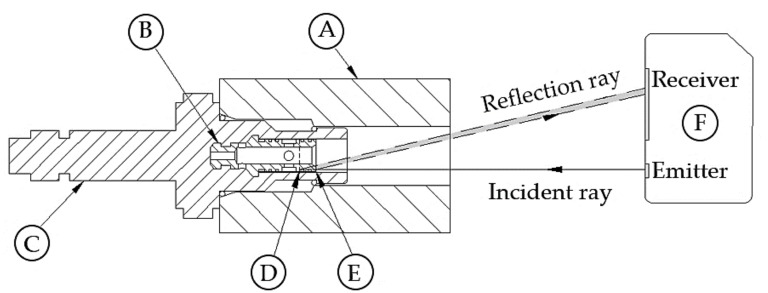
Principle of valve spool displacement measurement: A: Valve manifold; B: Spool; C: Iron core (and plunger, which is not shown); D and E: Spool upper and lower limits of travel; and F: Laser triangulation sensor.

The goal of this experiment is to identify the correlation between L and $x_{\delta }$,where L is determined from coil current alone, but $x_{\delta }$ is done by using an externally positioned laser triangulation sensor (LTS). The principle of the LTS in $x_{\delta }$ measurement is shown in Figure 4. A 655-nm wavelength incident ray emitted by an LTS hits the top land of the valve spool and is reflected back to the charge coupled device (CCD) on the receiving end. When the spool translates along the incident direction of the beam, the centroid position of the reflect spot on the CCD translates accordingly. By extracting the spot displacement information from the CCD, the corresponding displacement signal can be calculated based on the Pythagorean theorem.

### 3.2. Testbed

**Figure 5:**
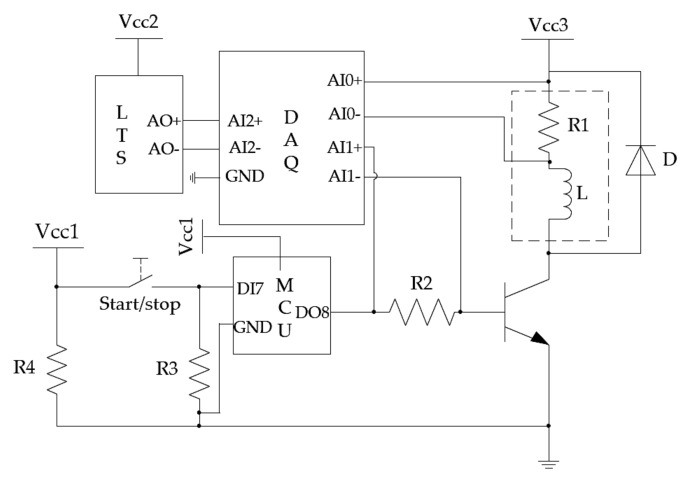
Circuit diagram of measurement and control system.

**Figure 6:**
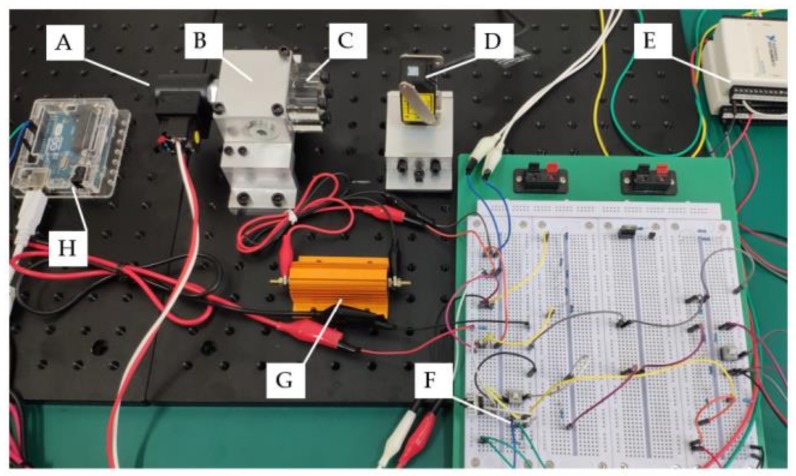
Photo of valve spool optical measurement system: A: Solenoid on–off valve; B: Aluminum valve manifold; C: Acrylic sight window (only installed during oil-immersed experiment); D: Laser triangulation sensor; E and F: Data acquisition and control; G: High-power electrical resistance; H: Micro-controller unit.

The testbed has four functions: (1) control of the solenoid on–off valve’s state (open or closed), (2) coil excitation and measurement of voltage-current in the drive circuit, (3) spool displacement measurement using LTS, and (4) data acquisition using a data acquisition board (DAQ). A circuit diagram of the testbed is shown in Figure 5. Vcc1 to Vcc3 are voltage supplies for the microcontroller unit (MCU), the LTS, and the solenoid, which are 5 V, 12 V, and 27 V (25.6 V on the coil), respectively. The MCU in the circuit is an Arduino Uno, which outputs constant 4.6 V to DO8 to switch on the transistor if a button-push-release action is detected at DI7, and 0V if next button action is registered. The purposes of resistors R1 (5 Ω), R2 (65 Ω) are to sense the current in the solenoid coil and the base current of the transistor, respectively. R3 (2 kΩ) is to positively bias the transistor against the output of the MCU, R4 (5 kΩ) serves as a current limiter, to protect the voltage source Vcc1. The sensed solenoid coil current via R1, the control single via R2, and the spool displacement from LTS are sampled by the DAQ’s analogue input channels from AI0 to AI2. The specifications of LTS used in this experiment are shown in Table 1. To ensure accurate measurement, sensor calibration to standard length blocks is performed over the intended spool measurement range prior to test. And a photo of the testbed is shown in Figure 6.

### 3.3. Dynamic Test Procedures

During the dynamic process of energizing and deenergizing of the solenoid valve, synchronous real-time acquisition of spool displacement, coil and drive current was performed. The programming of the MCU allowed that the solenoid coil was energized for 1.5 s and deenergized for 1.5 s. The acquisition started after the system was warmed up, i.e., the solenoid was cycled for 10 min to avoid temperature drift. In a multiple-cycle test, the acquisition time was 10 s and the sampling frequency was 10 kHz.

## 4. Results and Discussions

### 4.1. Data Acquisition and Preprocess

**Figure 7:**
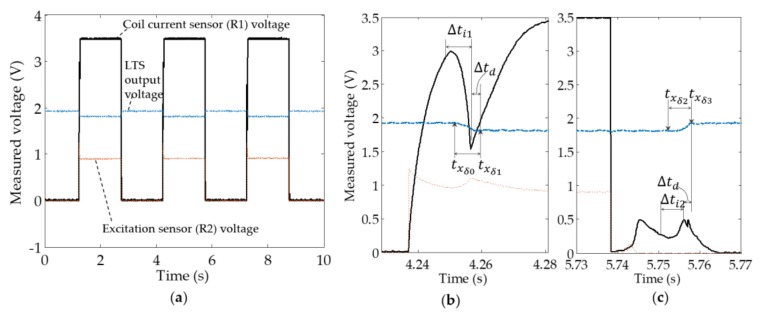
Multiple-cycle acquired data: (**a**) Illustration of multiple on–off cycles; (**b**) Obtaining of the desired section of data during air gap closing; (**c**) Obtaining of the desired section of data during air gap opening.

Based on the procedures discussed in Section 3.3, the test results of the solenoid valve in continuous on–off switching operation were recorded through the data acquisition system, as shown in Figure 7a. Before the calculation of the solenoid inductance, preprocess of data was performed to obtain the interested sections of spool displacement ($x_{d}$) and electrical current (i) data, when the solenoid was transitioning. This was done by locating the starting ($t_{x_{\delta 0}}$) and finishing ($t_{x_{\delta 0}}$) time stamps in the LTS signal when the valve was transitioning to open (air gap reducing to zero) as in Figure 7b; a selected section of spool displacement signal was $x_{d}\left(t_{x_{\delta 0}},t_{x_{\delta 1}}\right)$. According to the specification of the LTS from the manufacturer (in Table 1), it shows that the processing circuit of the LTS induces a $∆t_{d}=1/0.66{\;\mathrm{kHz}}^{-1}$ time delay from the measurement to signal output. The measured LTS signal, $x_{d}\left(t\right)$, shifted to $x_{d}\left(t-∆t_{d}\right)$ for delay compensation. The respective electrical current signal was selected to be $i\left(t_{x_{\delta 0}}-∆t_{d},t_{x_{\delta 1}}-∆t_{d}\right)$. The sections of data when the valve was transitioning to closed (air gap increasing from zero) can be obtained similarly (i.e., $i\left(t_{x_{\delta 2}}-∆t_{d},t_{x_{\delta 3}}-∆t_{d}\right)$) as in Figure 7c.

### 4.2. Calculation of $\hat{L}$

**Figure 8:**
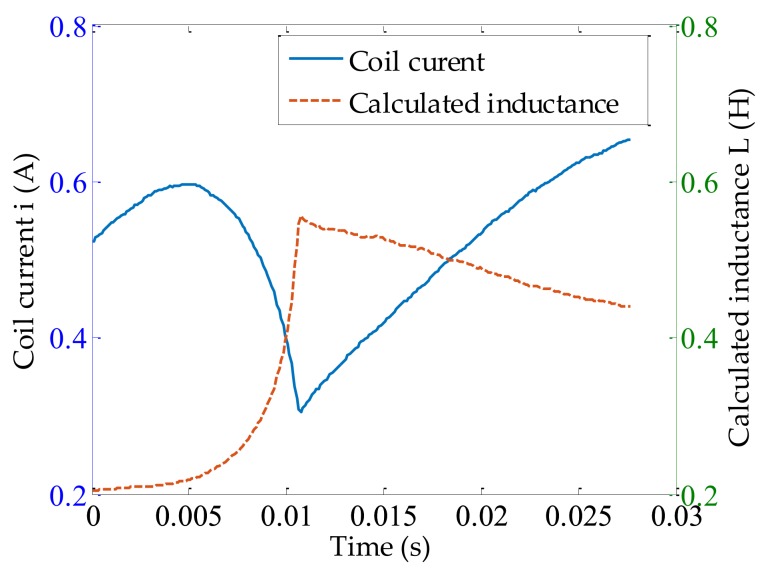
Calculated inductance ($\hat{L}$) as a function of measured current (i ) during air gap decreasing (solenoid is being energized).

To calculate $\hat{L}$ using the measured coil current in Equation (9), an initial condition of $L_{0}$ was required, which can be solved using Equation (12) through a grid search. But for the sake of calculation time, a range of search needed to be set. In Section 2.3, it was found that the grid search should be within $\left(L_{0}^{L}>0,L_{0}^{U}<\frac{\mu_{0}SN^{2}}{x_{\delta }\left(t\right)}\right)$. Based on the parameters of the on–off solenoid used in Table 2, the lower bound was found to be $L_{0}^{U}<\frac{\mu_{0}SN^{2}}{x_{\delta }\left(t\right)}=0.33\;H$. Then Equation (12) was solved within $\left(L_{0}^{L},L_{0}^{U}\right)$, and the calculated initial inductance value was 0.24 H. With an initial condition of $L_{0}$, the inductance within $∆t_{i}$ can be determined, the calculated $\hat{L}$ when the solenoid was energized is shown in Figure 8.

### 4.3. Determination of $L^{-1}$

**Figure 9:**
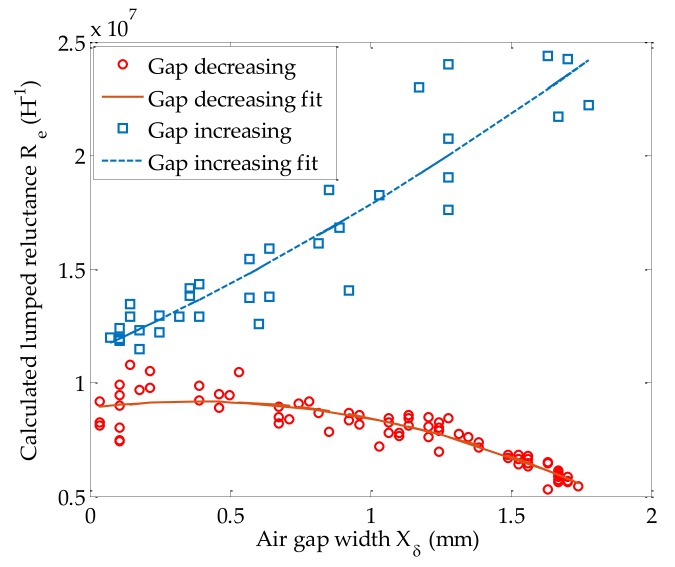
Relationship between $x_{\delta }$ and $R_{e}$ when the air gap increases or decreases.

The inverse relationship $x_{\delta }\left(t\right)=L^{-1}\left(x_{\delta },t\right)$ can be obtained as follows: First, the unknown $R_{e}\left(x_{\delta }\right)$ is solved by substituting the calculated inductance ($\hat{L}$) and the LTS measured air gap width ($x_{\delta }$) into Equation (8). Then, by performing a second order least square fit of the calculated $R_{e}$ to $x_{\delta }$ when the air gap is increasing or decreasing, two quadratic equations are found:

(13) $$R_{e}=k_{1j}x_{\delta }^{2}+k_{2j}x_{\delta }+k_{3j},$$

where the coefficients ($k_{ij}$) are the elements found in the matrix in Equation (14), subscript j denotes the air gap state to be decreasing (j=1) or increasing (j=2). Note that the relationship between $x_{\delta }$ and $R_{e}$ is only valid for this particular solenoid model used in this work. However, through a similar approach, this relationship can be obtained for other solenoid models. Figure 9 demonstrates Equation (13) for the cases when the air gap is increasing or decreasing.

(14) $$K=\left[\begin{array}{cc} -2.0\times 10^{12} & 9.4\times 10^{11}\\ 1.5\times 10^{9} & 5.6\times 10^{9}\\ 8.9\times 10^{6} & 1.1\times 10^{7} \end{array}\right]\begin{array}{c} m^{-2}H^{-1}\\ m^{-1}H^{-1}\\ H^{-1} \end{array},$$

Substituting Equation (13) into Equation (8), $R_{e}$ is eliminated:

(15) $$k_{1j}x_{\delta }^{2}+\left(k_{2j}+\frac{1}{\mu_{0}S}\right)x_{\delta }+k_{3j}-\frac{N^{2}}{\hat{L}}=0,$$

Using the root formula for a quadratic function, an explicit model between $x_{\delta }$ and $\hat{L}$ is found in Equation (16). Only the positive root of the two is selected due to the physical constraint ($x_{\delta }\geq 0$). The air gap width ($x_{\delta }$) is now explicitly defined using the calculated inductance of the coil ($\hat{L}$) alone. Since $\hat{L}$ is directly solved from Equation (9) using measured coil current (i). One can simply solve the air gap width from the coil current algebraically.

(16) $$x_{\delta }=L^{-1}=\frac{1}{k_{1j}}\left(\sqrt{{\left(k_{2j}+\frac{1}{\mu_{0}S}\right)}^{2}-4k_{1j}(k_{3j}-\frac{N^{2}}{\hat{L}})}-k_{2j}-\frac{1}{\mu_{0}S}\right),$$

### 4.4. Performance of Inductance Model

**Figure 10:**
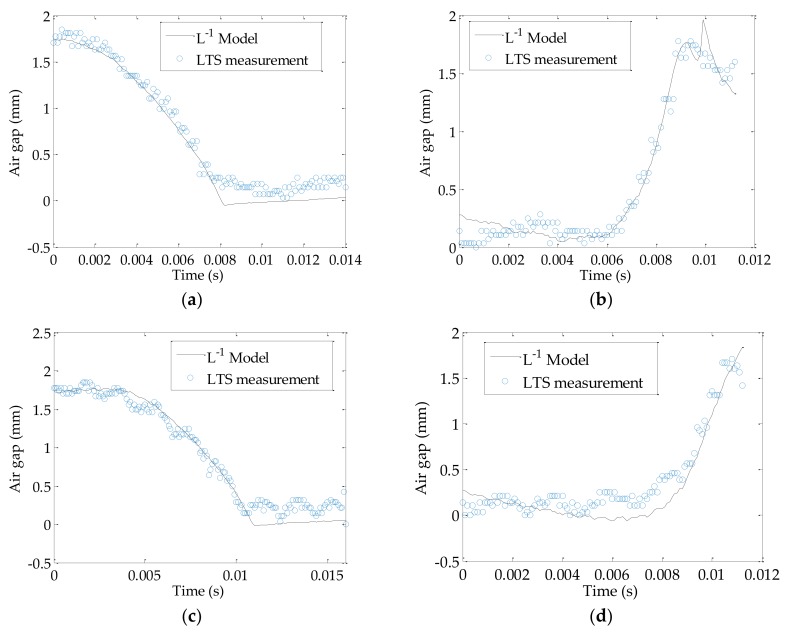

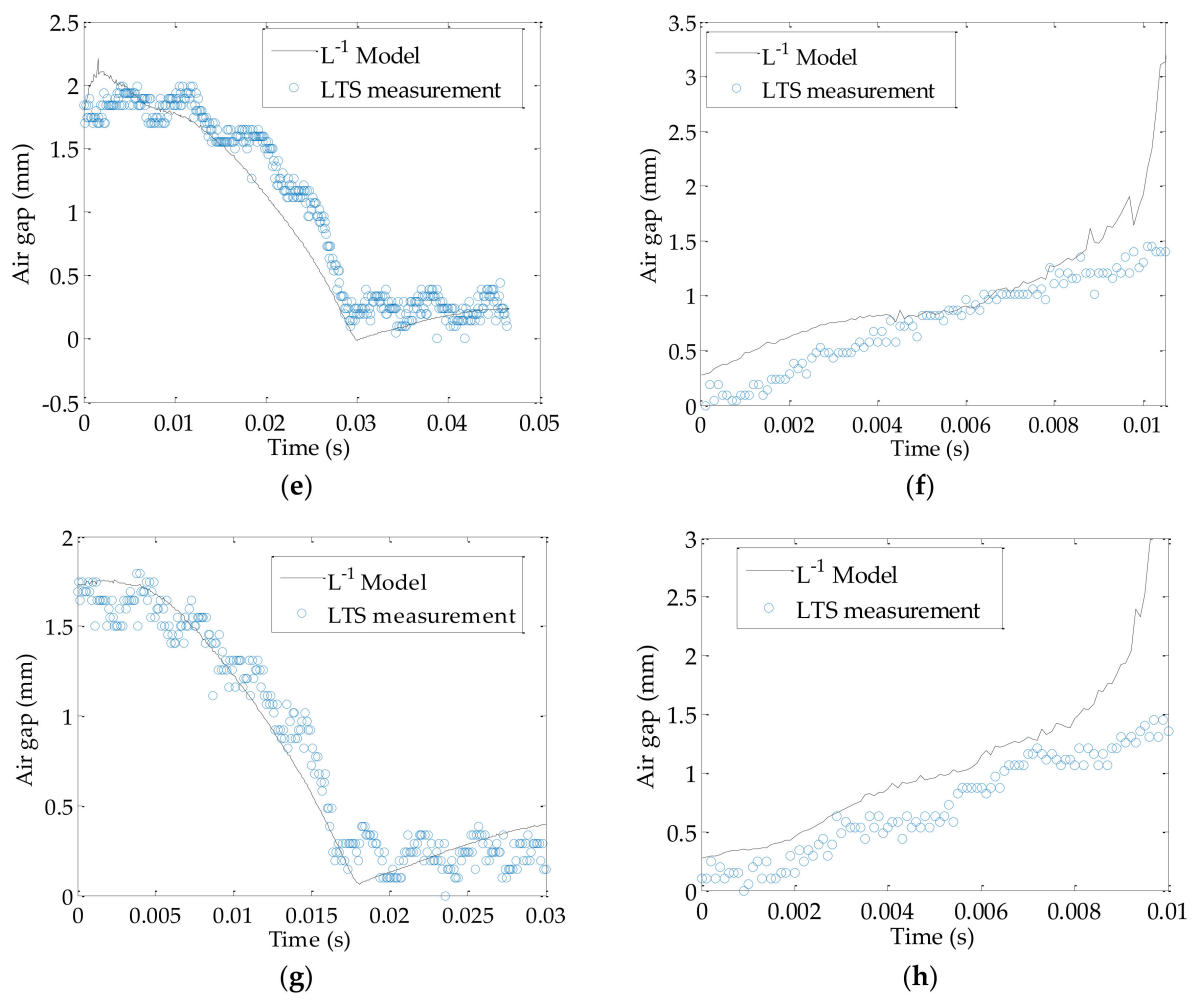
Performance of the model: (**a**) Valve is energized (air gap decreasing) in atmosphere under 25.6 V; (**b**) Valve is deenergized (air gap increasing) in atmosphere from 25.6 V; (**c**) Valve is energized in atmosphere under 23.5 V; (**d**) Valve is deenergized in atmosphere from 23.5 V; (**e**) Valve is energized in hydraulic oil under 26.3 V; (**f**) Valve is deenergized in hydraulic oil from 26.3 V; (**g**) Valve is energized in hydraulic oil under 29.0 V; (**h**) Valve is deenergized in hydraulic oil from 29.0 V.

The inductance model (Equation (16)) is tested and compared to the LTS measurement in Figure 10. In the experiment, the $L^{-1}$ model trialed in multiple operating conditions, i.e., the solenoid valve spool lubrication states (open in air or immersed in hydraulic oil) and coil drive current. It is seen that the $L^{-1}$ model compares well with the LTS measurement when the valve is in atmosphere, the predicted air gap width generally lies within the LTS measurement, during the increasing or decreasing of the air gap width, the error is within ±7%. The maximum deviation occurs nears the elbows of the curve, which is at 0.008 s of Figure 10a and 0.01 s of Figure 10b. The deviations are due to the zero-crossing of the time derivative of the current at those locations, which cause significant change in $\hat{L}$ when its magnitude is small. Since $\hat{L}$ is in the denominator in Equation (16), a rapid varying near-zero value would result in fluctuations in results. When the spool is driven under a lower potential as in Figure 10c,d, the $L^{-1}$ model is still able to estimate the air gap width with similar performance, despite more jaggedness in the LTS measurement, caused by the creeping motion of the spool due to lower coil drive potential.

Besides the experiments in open air, the manifold is also been refilled with ISO46 hydraulic oil and sealed with an acrylic sight glass, a similar setup as seen in Reference [10]. Since the travel distance of the laser spot is within 1.7 mm, the refraction of lights at the interfaces are not considered and directly lumped into a proportional coefficient (1.36) to evenly stretch the LTS results across 0—1.7 mm. Results in Figure 10e and 10f shows that even under similar driving voltage in Figure 10a and 10b, the air gap changes much slower in oil than air, which is expected since the viscosity and the flow pressure effects are much greater in oil. Comparing to air environment results, larger deviation of the $L^{-1}$ model from the LTS results are found not only at the elbows, but during the transitioning of the air gap width. The error under long transition time is considerably higher as in Figure 10e,f; the maximum error is around 5–57% depending on the temporal location. It is observed that if the duration of the air gap closing is similar to that in the air, by increasing the coil voltage to 29.0 V as in Figure 10g, in which case duration of the air gap transition to close becomes on par with Figure 10a,c, so does the performance of the predicted air gap width using the $L^{-1}$ model. In addition, the deenergized results in Figure 10h do not demonstrate significant difference from Figure 10f; both take an expectedly longer time to reopen the air gap compared to the cases in air due to viscous effects, and both diverge after the air gap is greater than 1.3 mm. In all, the findings indicate that there are time-dependent terms in the $L^{-1}$ model that have not been included yet, and based on the experimental results, the performance of the $L^{-1}$ model could deteriorate when the transition time of the valve is longer than 30 ms. However, since most industrial on–off valves operate around 10 to 40 ms, the issue of the unmodeled electromagnetic dynamics would only have noticeable effects on the estimation performance if the valve operates near the upper bound of the transition time, but the adverse effects can always be reduced by increasing the coil potential as in Figure 10g. Furthermore, the impacts of the coil current measurement noise and the magnetic permeability of environment on the $L^{-1}$ model, though small, could be significant in some cases especially when the air gap is opening (coil deenergizing), since magnitude of the coil current is small, the noise could be significant due to a low signal-to-noise ratio. To address the aforementioned issues, in future work, the authors believe that by introducing magnetic saturation into the formulation of $R_{e}$, employing onboard signal filtration, and additional calibration of the system in a hydraulic oil environment, the calculation accuracy of the $L^{-1}$ model can be further improved.

## 5. Conclusions

In this work, an electromagnetic model using the calculated inductance of an on–off solenoid to solve for the varying air gap width is derived. A testbed was designed and built to measure the coil current via a sensing electrical resistor and air gap with a laser triangulation sensor. By obtaining the magnetic reluctance from outside the solenoid using the derived definition of the coil inductance, a relationship between the air gap and the outside magnetic reluctance was determined. In this way, the air gap can be explicitly expressed as an algebraic equation with respect to coil inductance. Initial results show the calibrated model can well predict the transition of an air gap of an on–off solenoid in atmosphere with an error under ±7%. The model was also tested under a hydraulic oil environment, and results indicate the model is capable of predicting the displacement of the valve spool. In addition, the trade-offs of the $L^{-1}$ model have been identified and discussed. In the future, the nonlinearities of light refraction and other unmodeled dynamics could be added to the model, further improving the accuracy.

## Figures and Tables

**Table 1:** Main parameters of the laser displacement sensor.

Measurement Range (mm)	Linearity (%)	Laser Wavelength (nm)	Beam Diameter (μm)	Response Frequency (kHz)
100 ± 35	±0.1	655	120	0.66

**Table 2:** On–off solenoid parameters.

Coil Turns	Magnetic Permeability of Air (H/m)	Plunger Outer Diameter (mm)	Air Gap Area (mm^2^)	Maximum Air Gap Width (mm)	Coil Electrical Resistance (Ω)
2259	4π × 10^−7^	11.18	88.39	1.7	36.67

**Figure A1:**
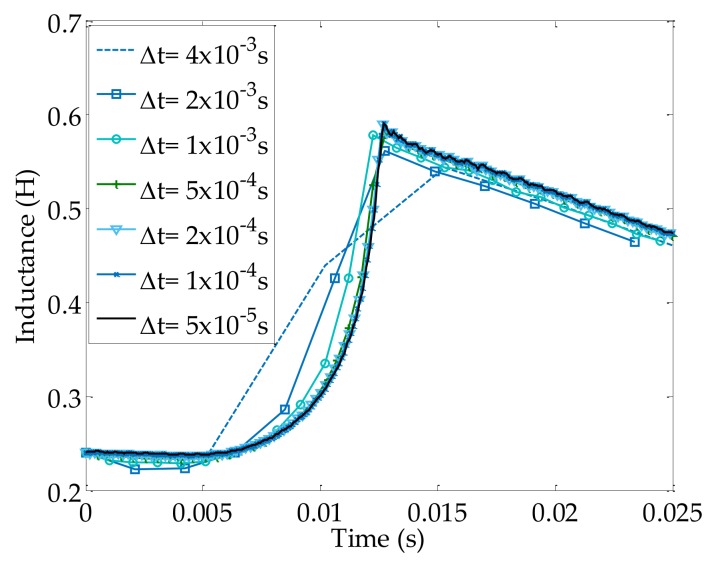
A convergence study of the effect of the time step size on the finite difference scheme proposed in Equation (9). The coil inductance was calculated from the measured coil current in the case when the valve was energized in atmosphere under 23.5 V.

## Appendix A. Convergence Study

The accuracy of the calculated coil inductance is crucial to the effective estimation of spool displacement. To ensure the convergence of the proposed finite difference scheme in Equation (9), seven different values of time step size were tested and the resulting coil inductance magnitudes are demonstrated in Figure A1. The calculated coil inductance converges as the grid size decreases. When ∆t is less than 5 × 10^−4^ s, the maximum deviation from the converged value (∆t= 5 × 10^−5^ s) is less than 4%. For the time step size selected in this work, ∆t= 1 × 10^−4^ s, the maximum deviation is less than 0.5%, which is deemed to have negligible impact on the performance of the $L^{-1}$ model.
